# Electrophysiological and Structural Remodeling of the Atria in a Mouse Model of Troponin-I Mutation Linked Hypertrophic Cardiomyopathy: Implications for Atrial Fibrillation

**DOI:** 10.3390/ijms22136941

**Published:** 2021-06-28

**Authors:** Wei-Wen Lim, Melissa Neo, Shivshankar Thanigaimani, Pawel Kuklik, Anand N. Ganesan, Dennis H. Lau, Tatiana Tsoutsman, Jonathan M. Kalman, Christopher Semsarian, David A. Saint, Prashanthan Sanders

**Affiliations:** 1 Centre for Heart Rhythm Disorders, South Australian Health and Medical Research Institute (SAHMRI), University of Adelaide and the Royal Adelaide Hospital, Adelaide, SA 5000, Australia; lim.wei.wen@nhcs.com.sg (W.-W.L.); melissa.neo@outlook.com (M.N.); shiv.thanigaimani@jcu.edu.au (S.T.); p.kuklik@uke.de (P.K.); anand.ganesan@flinders.edu.au (A.N.G.); dennis.lau@adelaide.edu.au (D.H.L.); 2 National Heart Research Institute Singapore, National Heart Centre Singapore, Singapore 169609, Singapore; 3 Programme in Cardiovascular and Metabolic Disorders, Duke-National University of Singapore Medical School, Singapore 169857, Singapore; 4 The Queensland Research Centre for Peripheral Vascular Disease, College of Medicine and Dentistry and The Australian Institute of Tropical Health and Medicine, James Cook University, Townsville, QLD 4811, Australia; 5 Department of Cardiology, Asklepios Klinik St. Georg, 20099 Hamburg, Germany; 6 Department of Cardiovascular Medicine, Flinders Medical Centre, Bedford Park, SA 5042, Australia; 7 Agnes Ginges Centre for Molecular Cardiology, Centenary Institute and the University of Sydney, Camperdown, NSW 2050, Australia; tatiana.tsoutsman@sydney.edu.au (T.T.); c.semsarian@centenary.org.au (C.S.); 8 Department of Cardiology, Royal Melbourne Hospital, Faculty of Medicine, Dentistry, and Health Sciences, University of Melbourne, Parkville, VIC 3010, Australia; jon.kalman@mh.org.au

**Keywords:** hypertrophic cardiomyopathy, atrial fibrillation, electrophysiology, histology, mice

## Abstract

Hypertrophic cardiomyopathy (HCM) is an inherited cardiac disorder affecting one in 500 of the general population. Atrial fibrillation (AF) is the most common arrhythmia in patients with HCM. We sought to characterize the atrial electrophysiological and structural substrate in young and aging Gly203Ser cardiac troponin-I transgenic (HCM) mice. At 30 weeks and 50 weeks of age (*n* = 6 per strain each group), the left atrium was excised and placed on a multi-electrode array (MEA) for electrophysiological study; subsequent histological analyses and plasma samples were analyzed for biomarkers of extracellular matrix remodeling and cell adhesion and inflammation. Wild-type mice of matched ages were included as controls. Young HCM mice demonstrated significantly shortened atrial action potential duration (APD), increased conduction heterogeneity index (CHI), increased myocyte size, and increased interstitial fibrosis without changes in effective refractory periods (ERP), conduction velocity (CV), inflammatory infiltrates, or circulating markers of extracellular matrix remodeling and inflammation. Aging HCM mice demonstrated aggravated changes in atria electrophysiology and structural remodeling as well as increased circulating matrix metalloproteinases (MMP)-2, MMP-3, and VCAM-1 levels. This model of HCM demonstrates an underlying atrial substrate that progresses with age and may in part be responsible for the greater propensity for AF in HCM.

## 1. Introduction

Hypertrophic cardiomyopathy (HCM) is the most common inherited cardiac disease affecting at least 1 in 500 of the general population, with increased prevalence when accounting for modern advances in clinical and genetic diagnoses [1,2,3]. Despite this, a considerable proportion of HCM patients likely remain undiagnosed throughout their lifetime [1,3]. HCM is associated with significant adverse outcomes such as risks of sudden cardiac death often in young patients [4], whereas progressive heart failure and atrial fibrillation develops with age [5].

Atrial fibrillation (AF) is the most common rhythm disorder affecting patients with HCM and confers an added risk of developing heart failure, death, and stroke [1,5,6]. The overall prevalence of AF is postulated to be ≈22% in HCM patients [7,8], with increased left atrial (LA) size and age being major predictors for AF and stroke development [8]. AF prevalence in HCM is much greater compared to 2–4% in adults and up to 10–17% in those older than 80 years of age [9,10]. AF prevalence in HCM is not rare in younger patients (35% developed symptomatic AF at ≤50 years of age) [11], and it is associated with worse survival outcomes compared to AF-free HCM patients [7,12]. Interestingly, Siontis et al. (2014) demonstrated that AF was more common in non-obstructive HCM and associated with larger left atria, higher E/e’ ratios, and worse cardiopulmonary exercise tolerance, which may be suggestive of intrinsic atrial myopathy as a cause for AF. With the social and economic burden posed by AF being on the rise [13], there is an urgent need for targeted therapies of AF particularly in the HCM cohort where AF incidence occurs in younger patients with greater mortality risks as age progresses.

Underlying atrial myopathy forms the substrate for AF development [14]. However, there is little information and no evaluation of the atrial substrate predisposing to the development of AF in HCM. Therefore, it is of interest to identify the progression of the atrial substrate in HCM, which may allow for timely interventions to prevent or perhaps reverse atrial myopathy to reduce AF risk. The Gly203Ser cardiac troponin-I (TnI) transgenic mouse model of HCM overexpresses human disease-causing Gly203Ser mutation known to disrupt normal troponin complex interaction, resulting in abnormal calcium cycling, and it develops characteristic features of human HCM by 21 weeks [15]. A previous study by our group demonstrated slowed atria and atrioventricular conduction and depressed heart rate variability conserved with aging in the model [16]. In the current study, we investigated the atrial substrate in the mouse model, which may help explain AF development in HCM patients. In particular, we assessed the atrial structural and electrophysiological alterations and blood biomarkers in this model of HCM. Additionally, we evaluated the impact of aging on the HCM atria in these mice.

## 2. Results

### 2.1. Animal Characteristics

Detailed animal characteristics are presented in Table 1. There were no significant differences in body weight, heart rates, and blood pressures between HCM and control mice at both age groups, although significantly lower blood pressures were noted in aged mice as compared to young mice (85.8 ± 0.6 vs. 94.9 ± 0.4 mmHg; *p* < 0.001). HCM animals demonstrated a greater left ventricular and septal wall thickness compared to controls at both ages (both *p* < 0.0001) along with significant bi-atrial hypertrophy evident by the increased left and right atrial mass (*p* < 0.01 and *p* < 0.05 respectively) (Table 1). No significant age difference was observed in the aforementioned parameters between young and aged HCM mice.

### 2.2. Atrial Action Potential Duration and Effective Refractory Period

**Figure 1:**
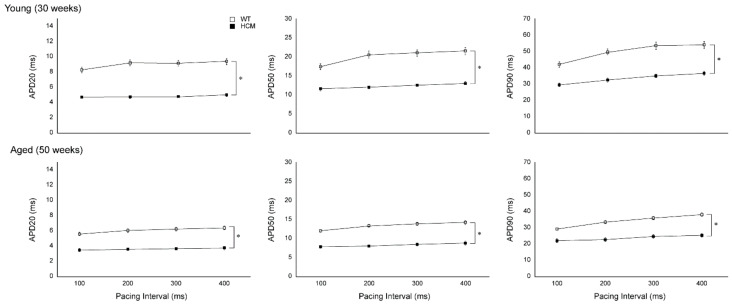
Action potential durations (APD) at 20% (APD20), 50% (APD50), and 90% (APD90) repolarization are reduced in both young and aged hypertrophic cardiomyopathy (HCM) atria. Young HCM mice atria demonstrated reduced APD20, APD50, and APD90 compared to control mice across all pacing cycle lengths tested. Similarly, aged HCM mice also showed reduced APD20, APD50, and APD90 compared to controls across all pacing cycle lengths tested. * *p* < 0.001.

Atrial action potential durations (APD) at 20% (APD20), 50% (APD50), and 90% (APD90) repolarizations were significantly reduced in young HCM mice compared to controls (APD20: 4.74 ± 0.07 vs. 8.93 ± 0.25 ms, APD50: 12.19 ± 0.32 vs. 19.94 ± 0.90 ms, APD90: 33.09 ± 1.5 vs. 49.31 ± 2.75 ms, respectively; all *p* < 0.001; Figure 1). The decreases in APD20, 50, and 90 were uniformly reduced in the young HCM mice compared to controls across all pacing cycle lengths tested and were conserved with aging (Figure 1).

**Figure 2:**
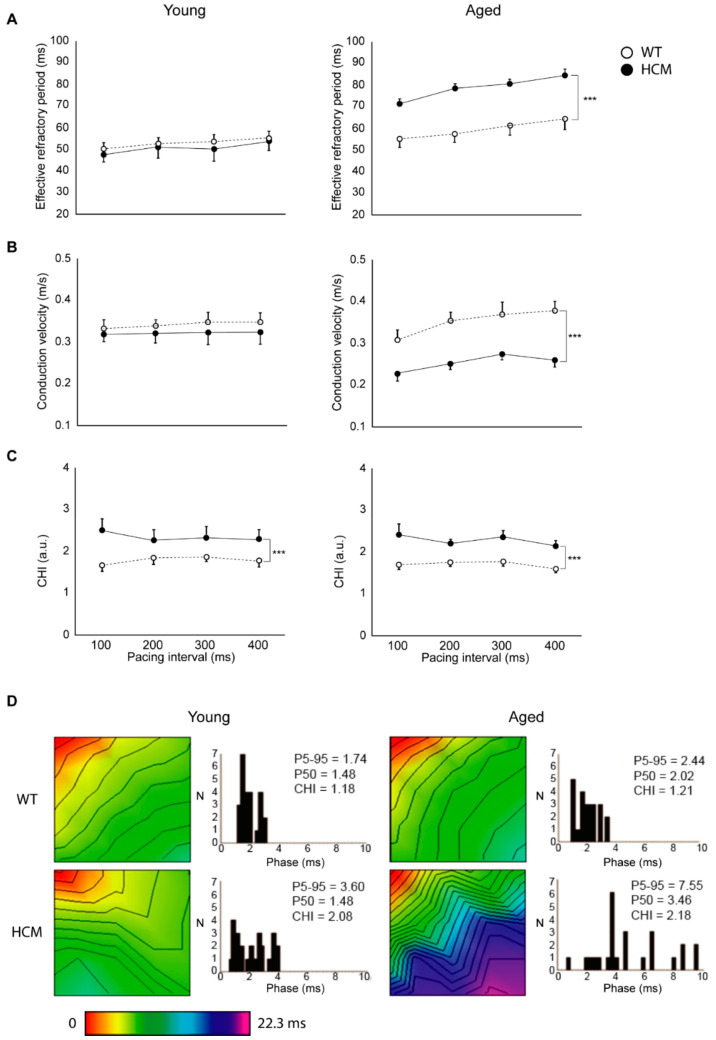
Electrophysiological changes in the HCM left atria (LA). ((**A**), left) Young HCM mice demonstrate unchanged effective refractory period (ERP), whereas aged mice (right) exhibit increased ERP across all pacing cycle lengths. ((**B**), left) Young HCM mice demonstrate unchanged conduction velocity (CV), whereas aged mice (right) exhibit decreased CV across all pacing cycle lengths. (**C**) Both young (left) and aged (right) HCM mice demonstrate increased conduction heterogeneity index (CHI) as compared to controls across all pacing cycle lengths. (**D**) Representative LA time maps and corresponding phase histograms of mice at 30 weeks and 50 weeks at 200 ms pacing. Phase histograms depict the number of episodes on the *y*-axis and the largest phase difference within each quadruplet of electrodes on the *x*-axis. Young HCM mice demonstrate unchanged total activation time but increased conduction heterogeneity progressing to slowed heterogeneous conduction in aged mice. Isochrones (black lines) are constructed at 1 ms intervals. Red indicates the start of the electrical impulse activation and pink represents the longest activation time with intermediate values color coded according to the time scale below. *** *p* < 0.001.

In contrast, we observed that changes in atrial epicardial ERPs were not similar to APD results. Atrial ERPs in the young HCM mice were not different from controls across all pacing intervals (51 ± 6 vs. 52 ± 3 ms, respectively, at a pacing interval of 200 ms; *p* = NS; Figure 2A). With aging, HCM mice demonstrated a significant prolongation of ERP across all pacing intervals (78 ± 4 vs. 57 ± 2 ms, respectively, at a pacing interval of 200 ms; *p* < 0.001). Additionally, a significant decrease in ERP was observed at faster pacing rates (400 vs. 100 ms pacing) in both HCM and control mice (HCM: 84 ± 5 vs. 71 ± 4 ms respectively; wild-type (WT): 64 ± 3 vs. 54 ± 2 ms respectively; both *p* < 0.01).

### 2.3. Atrial Conduction

Young HCM mice demonstrated unchanged conduction velocity (0.323 ± 0.023 vs. 0.341 ± 0.014 m/s respectively at 200 ms pacing; *p* = NS) but increased conduction heterogeneity (2.29 ± 0.27 vs. 1.88 ± 0.17 respectively at 200 ms pacing; *p* = 0.001) across all pacing cycle lengths as compared to age-matched controls (Figure 2B,C). In the older cohort, HCM animals demonstrated a reduction in conduction velocity (0.251 ± 0.012 vs. 0.353 ± 0.021 m/s respectively at 200 ms pacing; *p* = 0.001) and also greater conduction heterogeneity index (2.21 ± 0.10 vs. 1.76 ± 0.10 respectively at 200 ms pacing; *p* = 0.001) across all pacing cycle lengths compared to controls. Figure 2D shows the activation time maps and corresponding phase histograms from representative LA of HCM and control mice during S1 pacing at 200 ms.

### 2.4. Atrial Structural Remodeling

**Figure 3:**
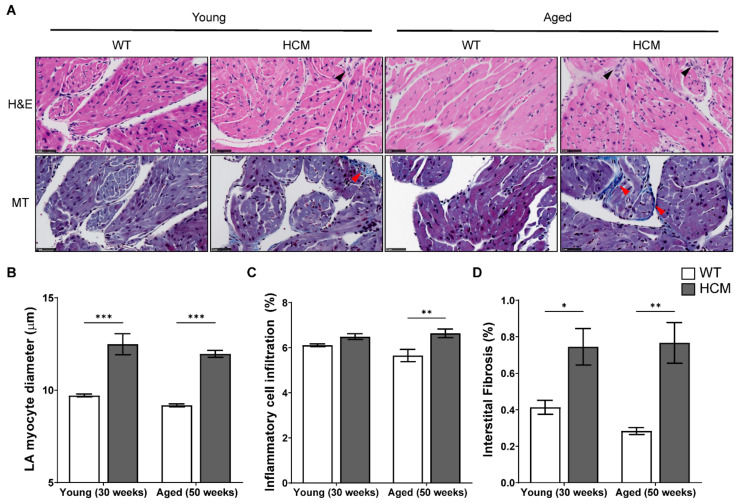
HCM mouse atria demonstrates myocyte hypertrophy, elevated inflammatory cell infiltrates, and interstitial fibrosis. (**A**) Representative photomicrographs of transverse sections of the LA stained with hematoxylin and eosin (H&E; top) and Masson’s trichrome (MT; bottom) in young and aged HCM mice demonstrated increased inflammatory cell infiltration (black arrowheads) and interstitial fibrosis (stained blue; red arrowheads) in the atrial myocardium as compared to controls. Scale = 50 µm. (**B**) Pooled data on LA cardiomyocyte diameter, (**C**) inflammatory cell infiltrate, and (**D**) atrial fibrosis across six independent animals per group. No significant age differences were observed. * *p* < 0.05, ** *p* < 0.01 and *** *p* < 0.001.

Representative photomicrographs of the left atrium in young and aged WT and HCM mice stained with H&E and Masson’s Trichrome are illustrated in Figure 3A. HCM mice atria demonstrates increased cardiomyocyte hypertrophy (both *p* < 0.001; Figure 3B), increased inflammatory cell infiltration (Young: *p* = NS; Aged: *p* < 0.001; Figure 3C), and increased interstitial fibrosis (Young: *p* < 0.05; Aged: *p* < 0.01; Figure 3D) as compared to controls. Additionally, we did not observe statistical differences between the age groups in any of the atrial structural parameters assessed.

**Figure 4:**
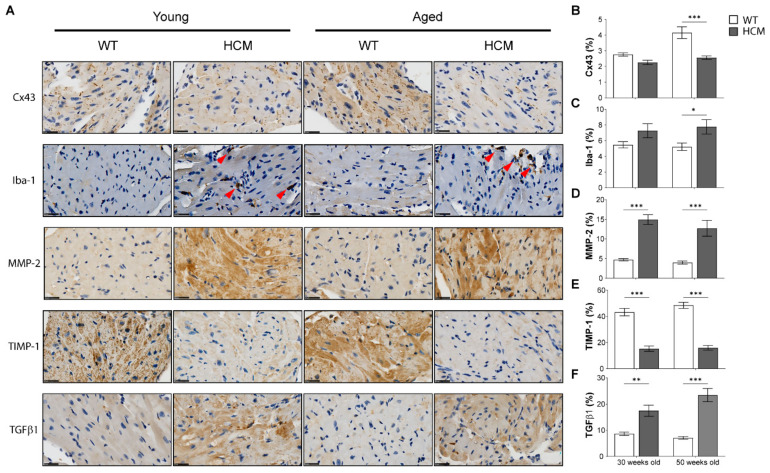
HCM mouse atria demonstrates altered connexin 43 (Cx43) expression, increased macrophage infiltration, and changes in extracellular matrix remodeling. (**A**) Representative photomicrographs of transverse sections of the LA stained for Cx43, ionized calcium binding adaptor molecule 1 (Iba-1) macrophage, matrix metalloproteinase-2 (MMP-2), tissue inhibitor of metalloproteinase 1 (TIMP-1), transforming growth factor beta 1 (TGFβ1) expression. Red arrowheads indicate Iba-1^+ve^ macrophages. Scale = 25 µm. Cumulative data on Cx43 (**B**), Iba-1 (**C**), MMP-2 (**D**), TIMP-1 (**E)**, and TGFβ1 (**F**) expressed as a percentage of total myocardium area in six independent samples per group. * *p* < 0.05, ** *p* < 0.01 and *** *p* < 0.001.

Representative photomicrographs of the left atrium in young and aged WT and HCM mice stained with connexin 43 (Cx43), ionized calcium binding adaptor molecule 1 (Iba-1), matrix metalloproteinase 2 (MMP-2), tissue inhibitor of metalloproteinase 1 (TIMP-1), and transforming growth factor beta 1 (TGFβ1) are presented in Figure 4A. Aged WT mice demonstrated enhanced Cx43 expression and lateralization consistent with normal aging [17], whereas Cx43 expression remained low in aged HCM atria (*p* < 0.001; Figure 4B). We observed an increased presence of Iba-1^+ve^ macrophages in the atrial myocardium in both young and aged HCM mice as compared to controls (*p* = 0.154 and *p* < 0.05 respectively; Figure 4C), which is consistent with our observations of an increase in inflammatory cell infiltration by H&E (Figure 2C) and suggestive of a pro-inflammatory profile in the HCM atria. Lastly, the atria of young and aged HCM mice demonstrated increased MMP-2 (both *p* < 0.001; Figure 4D), decreased TIMP-1 (both *p* < 0.001; Figure 4E), and elevated TGFβ1 expression (Young: *p* < 0.01, Aged: *p* < 0.001; Figure 4F). No significant differences were observed between young and aged HCM mice in the expression of the aforementioned immunostained markers.

### 2.5. Biomarkers of Extracellular Matrix Remodeling and Inflammation

**Figure 5:**
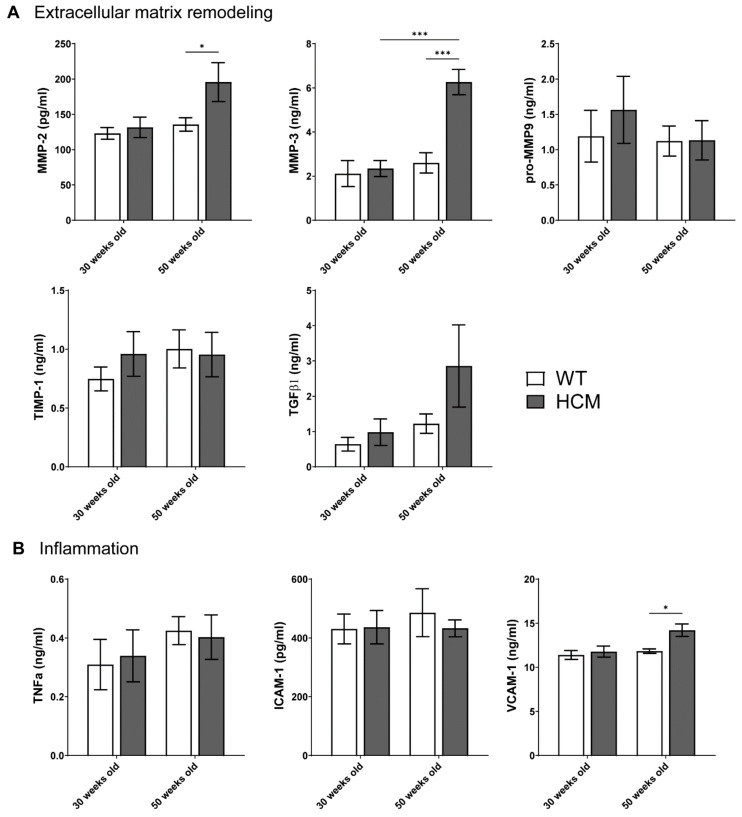
Circulating plasma biomarkers of extracellular matrix and inflammation. (**A**) Plasma levels of extracellular matrix biomarkers MMP-2, -3, pro-MMP-9, TIMP-1, and TGFβ1 in young and aged HCM mice compared to controls. (**B**) Plasma levels of inflammatory biomarkers tumor necrosis factor alpha (TNFα), intercellular adhesion molecule 1 (ICAM-1), and vascular cell adhesion molecule 1 (VCAM-1) in both young and aged HCM mice compared to controls. * *p* < 0.05 and *** *p* < 0.001.

To investigate biomarkers of extracellular matrix remodeling that may explain the elevated atrial fibrosis in the HCM atria, we assessed the plasma concentrations of MMP-2, MMP-3, pro MMP-9, TIMP-1, and TGFβ1 in HCM and control mice at both 30 and 50 weeks of age (Figure 5A). No significant changes in plasma levels of MMP-2, -3, pro MMP-9, TIMP-1, and TGFβ1 were observed in the young HCM mice as compared to controls. In contrast, significant elevations in circulating MMP-2 and MMP-3 were observed in aged HCM mice compared to age-matched controls (MMP-2: 208.0 ± 41.5 vs. 128.2 ± 7.4 pg/mL, MMP-3: 5.5 ± 0.7 vs. 2.0 ± 0.4 ng/mL; *p* < 0.05 and *p* < 0.01 respectively). Pro-MMP-9 and TIMP-1 levels were not statistically altered in the aged HCM mice as compared to controls. Plasma levels of TGFβ1, a biomarker of fibrosis, were elevated in aged HCM mice compared to controls, although this did not reach significance due to large variability (2.9 ± 1.2 vs. 0.6 ± 0.1 ng/mL; *p* = 0.130).

To investigate the increased inflammatory cell infiltration in the HCM atria, we assessed plasma concentrations of tumor necrosis factor alpha (TNFα), intercellular adhesion molecule 1 (ICAM-1), and vascular cell adhesion molecule 1 (VCAM-1) in both HCM and control mice at both ages (Figure 5B). Young HCM mice demonstrated unchanged concentrations of TNFα, ICAM-1, and VCAM-1 compared to controls. In contrast, plasma levels of VCAM-1 were elevated in the old HCM mice as compared to controls (13.9 ± 0.8 vs. 11.8 ± 0.3 ng/mL; *p* < 0.05), whereas concentrations of TNFα and ICAM-1 were not different from controls.

## 3. Discussion

Using an established mouse model of hypertrophic cardiomyopathy, this study characterizes the structural and electrophysiological remodeling of the atria that potentially predisposes to the development of AF. This study presents the following new information regarding the atrial remodeling in HCM:

1. Both atria were enlarged with an increase in atrial myocardial mass in young and aged HCM mice.
2. Marked atrial structural abnormalities with myocyte hypertrophy and fibrosis was observed in young HCM mice and aged HCM mice demonstrated myocyte hypertrophy, inflammatory cell infiltration, and fibrosis.
3. Electrophysiological abnormalities within the atria were observed from a young age with abbreviations in the action potential duration and an increase in conduction heterogeneity. However, abnormal refractoriness and conduction velocity were only observed with increasing age.
4. Aged HCM mice demonstrated increased circulating levels of extracellular matrix remodeling MMP-2, MMP-3, and inflammation VCAM-1, which were unaltered in the younger cohort.

**Figure 6:**
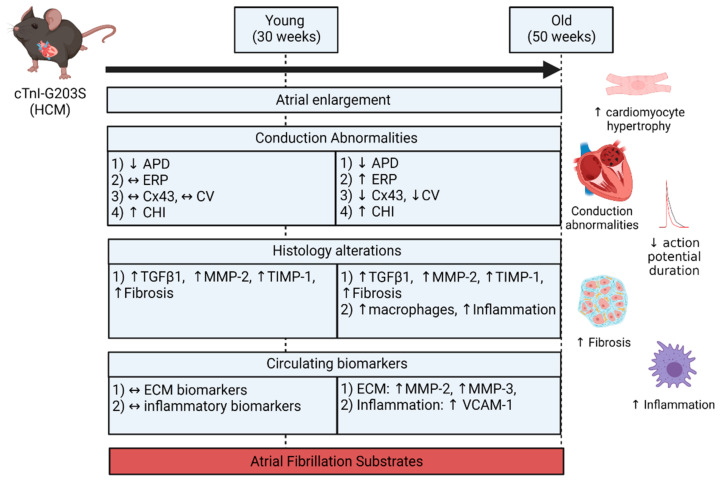
Summary of atrial conduction, histological, and circulating biomarker alterations in the troponin I (TnI) mutant mouse model of HCM. Created with BioRender.com on 17 June 2021.

These observations demonstrate that HCM is associated with an abnormal atrial substrate that is further potentiated with increasing age (summarized in Figure 6). This milieu of abnormalities may in part result in the increased burden of AF in HCM patients with age.

### 3.1. Atrial Substrate Predisposing to AF

Rapid atrial activation, either through artificial AF maintenance [18] or rapid atrial pacing [19], has been demonstrated to induce electrical alterations such as decreased atrial refractoriness, slowed and heterogeneous conduction, thereby promoting AF, namely termed ‘AF begets AF’. Whilst this may explain the progression of paroxysmal to permanent AF, it does not adequately explain the substrate predisposing to AF development. Alternately, Li et al. first demonstrated that experimental congestive heart failure in dogs modifies the atrial substrate by promoting atrial interstitial fibrosis, resulting in regional slowed and heterogeneous conduction and thereby increasing the inducibility and duration of AF [20]. These findings were replicated in humans with congestive heart failure [21]. Furthermore, atrial fibrosis and conduction abnormalities were not restored despite the reversal of congestive heart failure [22], suggesting that the possibility of an atrial substrate that can support AF propagation remains. Localized conduction abnormalities have also been observed implicating AF in the setting of atrial ischemia [23], obesity [24], obstructive sleep apnea [25], hypertension [26], cardiomyopathy [27], and binge drinking [28]. Likewise, slower and heterogeneous atrial conduction associated with increased age was observed in the current model of HCM. However, contrasting to the notion of ‘AF begets AF’ where atrial refractoriness is known to be progressively reduced to drive the stability of AF [18], atrial refractoriness has been variably reported to be either decreased [23], unchanged [24,25], or increased [26,27], depending on the underlying heart disorder. This may perhaps suggest that atrial refractoriness is disease-dependent and unlikely a defined component in the substrate for AF.

An interesting finding in the current study was the dissociation between APD and ERP in aged HCM mice, which is often assumed to be correlated. Similar findings of shortened APD have been commonly reported with aging, whereas ERP observations have been conflicting [29]. Action potential morphology depends on the geometry, boundary, and direction of impulse propagation [30]. Structural heterogeneity, in computational models, modulates the ERP with little effects on APD [31]. The discordant APD-ERP findings here may reflect regional heterogeneity in ERP distribution and the non-linear correlation between APD and ERP.

### 3.2. Atrial Remodeling in HCM

Most transgenic mouse models to date have replicated a combination or all of the human HCM phenotype; mainly ventricular myocardial fibrosis, hypertrophy, myofibrillar disarray, and left ventricular dysfunction [15,32,33,34,35]. Atrial enlargement has occasionally been observed in some HCM mice models [32,34] and not others [35]. However, in-depth assessment of atria electrophysiological and structural substrates was not investigated. Atrial enlargement is a common structural trait in experimental models of heart failure [21], mitral valve disease [33], and ventricular cardiomyopathy [32]. Whilst significant bi-atrial enlargement with slowed and heterogeneous atrial conduction was observed in our model of HCM, we did not observe any inducible AF. One possible explanation may be the small chamber size of the murine atrium ex vivo that may preclude the regeneration of re-entrant circuits and waves that characterize larger atria [36,37]. Alternatively, increased refractoriness in the aged HCM mice may counteract the slower conduction by normalizing the electrical impulse wavelength that accounts for re-entrant activity [38]. Nonetheless, our observations agree with human studies demonstrating age-associated increased refractoriness [39]. Reduced atrial APD recorded from the atrial endocardium in HCM mice may reflect spatial and transmural heterogeneity in the atrium and may potentially serve as a proarrhythmic determinant [40].

Fibrosis and inflammation are fundamental hallmarks of atrial structural remodeling contributing to a sustained substrate for AF progression. Interstitial fibrosis and inflammatory infiltration were key histological findings in the current study. An increase in spatial heterogeneity in atrial conduction may be explained by the development of regional fibrosis resulting in electrical uncoupling of adjacent myocytes. Myocardial electrical impulse propagation is governed by low-resistance connexin channels located in the gap junction of adjacent myocytes, which can be disrupted by fibrosis interrupting inter-myocyte coupling [41]. Cx43 was observed to be reduced in the HCM atria and conserved with aging. As a result, electrical propagation is reduced, leading to focal areas of slowed conduction and block, contributing to the development of an AF substrate.

Alterations in plasma matrix metalloproteinases (MMPs) and tissue inhibitors of metalloproteinases (TIMPs), key proteins involved in the homeostasis of collagen turnover and resultant fibrosis, have been demonstrated in adverse structural and functional manifestations of ventricular remodeling in HCM [42,43] and aging [44]. In atrial biopsies, the downregulation of TIMP-2 and upregulation of MMP-2 correlates with the development of sustained AF in patients with cardiomyopathy and heart failure [45]. Furthermore, plasma levels of MMP-9, MMP-3, and TIMP-4 independently predict AF recurrence in post-cardioversion patients [46]. Our observations in MMPs/TIMP-1 alterations specifically in aging TnI mutant mice may reflect an established disease state, where they previously were found to be indistinguishable from non-transgenic mice prior to significant phenotype development [47]. Additionally, we observed increased plasma TGFβ1, a pivotal inducer of cardiac fibrosis in HCM [48], although this did not reach significance due to variability, which may reflect the severity of disease.

Inflammation is linked to various pathological processes including oxidative stress and fibrosis that in itself promotes AF substrate formation [49,50]. Mast cells, neutrophils, and macrophages secrete pro-inflammatory cytokines such as TNFα that induces adhesion molecules ICAM-1 and VCAM-1 induction [51]. Schultz et al. have previously demonstrated that circulating levels of ICAM-1 were significantly greater in AF patients compared to subjects with supraventricular tachycardia and non-AF controls [52]. Therefore, ICAM-1 levels in our study may be unchanged, as our model did not demonstrate inducible AF. Macrophages are another source of inflammatory cell infiltration, which has previously been shown to drive remodeling in HCM [53]. Likewise, we found increased Iba-1^+ve^ macrophages in the HCM atria albeit without significant changes in circulating TNFα and ICAM-1 levels. Contrastingly, an increase in circulating VCAM-1 levels, an endothelial promoter of monocyte and lymphocyte recruitment and inflammation marker recently associated with AF [54], was observed in aged mice with HCM.

### 3.3. Potential Mechanisms of HCM-Induced Atrial Remodeling

One proposed mechanism behind the increased AF prevalence in HCM lies in diastolic dysfunction and/or mitral regurgitation that increases LA pressure, leading to chronic dilatation and increased size and in turn leads to atrial electrical remodeling [55]. Left ventricular myocardial fibrosis impairs ventricular function and is associated with greater AF incidence in HCM patients [56].

An alternative hypothesis may be the coexistence of atrial myopathy, which may determine LA dysfunction and the development of AF due to a fragmentation of atrial conduction; this hypothesis is indirectly supported by observations of individuals with specific Arg633His *MYH7* mutations demonstrating less cardiac hypertrophy as compared to other *MYH7* mutations but a higher risk of developing AF [57]. Areas of regional fibrosis in the atria can result in electrical uncoupling of adjacent cardiomyocytes, leading to a fragmentation of atrial propagation, resulting in areas of conduction slowing and block, and increased conduction heterogeneity, ultimately promoting focal and macro-re-entry AF [41].

### 3.4. Study Limitations

This study documents atrial remodeling associated with TnI mutant HCM mouse to evaluate the substrate predisposing to AF and may not necessarily replicate other HCM sarcomeric protein mutations such as *MYH7* or *MYBPC3*. Although significant atrial myopathy has been identified in our current model of HCM, we are unable to delineate if the atrial remodeling was a result of primary atrial myopathy or secondary to ventricular dysfunction in the mouse ages chosen, which may be better understood by a longitudinal study with more time points depicting pre-, during, and end-stage ventricular hypertrophy in HCM. Notably, we did not document AF incidence in an ex vivo electrophysiology study, which may be better assessed by telemetric electrocardiography.

### 3.5. Conclusions

Evidence suggests that atrial myopathy plays a crucial role in providing a substrate that predisposes AF development [14]. However, the delineation of the pathophysiological traits in the diseased atria in the setting of HCM has not been adequately characterized, although atrial size, fibrosis, and inflammation are all likely perpetrators for AF development. Atrial remodeling is implicated in the pathogenesis of AF and may explain the increased AF prevalence in HCM with increasing age. Pulmonary vein isolation, coupled with the targeted complex fractionated atrial electrogram ablation which may occur in areas of fibrosis, conduction slowing, or block [58], has recently been shown to be effective in maintaining long-term rhythm control in HCM patients [59]. Therefore, early modulation of the atrial electrophysiological and structural changes may represent new therapeutic approaches in the early treatment and prevention of AF in HCM.

## 4. Materials and Methods

### 4.1. Animal Model 

We used the Gly203Ser cardiac troponin-I transgenic mouse model as previously described [15]. Mice were bred on the C57BL/6 genetic background and have been shown to have a normal life span. Transgenic (HCM) mice with the mutation have been known to develop ventricular phenotypic hallmarks of HCM by 21 weeks of age. A total of 12 HCM animals were studied in 2 groups at age 30 and 50 weeks of age. As a control group, non-transgenic C57BL/6 (control) mice were used, which have been previously demonstrated to be indistinguishable from transgenic wild-type overexpression mice [15]. A total of 12 control animals were studied in the 2 groups at age 30 and 50 weeks of age.

HCM and control mice were bred by crossing an HCM female with a wild-type male mouse and were tail genotyped at 2–3 weeks of age for identification. Then, mice were allocated to the two groups. Mice were housed at controlled temperature (24 °C) and lighting (12-h light–dark cycles) with free access to standard chow and water ad libitum. All experiments were approved by the University of Adelaide Animal Ethics Committee and conducted in accordance with the Australian Code of Practice for the Care and Use of Animals for Scientific Purposes.

### 4.2. Heart Rate and Blood Pressure 

Non-invasive tail-cuff systolic blood pressures and heart rates of mice were measured using the NIBP controller (AD Instruments, Australia) under anesthesia with intraperitoneal injection (75 mg/kg ketamine (100 mg/mL) and 0.5 mg/kg medetomidine (1 mg/mL)) immediately prior to sacrifice. Depth of sedation was checked periodically for paw- and tail-pinch reflexes. Reported values are derived from a mean of 3 consecutive readings.

### 4.3. Action Potential Duration and Multi-Electrode Array Electrophysiology Study 

Simultaneous action potential recordings were recorded during electrophysiology studies as previously described [60]. At the respective endpoints (30 or 50 weeks of age) and following anesthesia, a single intraperitoneal injection of heparin (2-IU/g body weight) was administered prior to blood sampling. A midline thoracotomy incision was performed, and the heart was promptly removed and rinsed with ice-chilled bicarbonate buffer solution. The left atrium was carefully dissected from the heart and the epicardial surface was placed onto the multi-electrode array (MEA) housed within a flow chamber irrigated with bicarbonate buffered solution (in mM: 130 NaCl, 4 KCl, 0.6 MgCl_2_, 24 NaHCO_3_, 1.2 NaH_2_PO_4_, 12 D-glucose, 1.5 CaCl_2_) maintained at 37 °C and pH 7.4 when aerated with 95% oxygen and 5% carbon dioxide. The MEA was custom made, consisting of 6 × 6 electrodes of 0.1 mm diameter and 0.5 mm inter-electrode distance yielding a total of 25 bipolar electrograms. Electrograms from the MEA were digitally sampled at a rate of 2 KHz and filtered from 10 to 500 Hz. These were recorded to commercially available computerized recording systems (LabSystem Pro, Bard Electrophysiology, Lowell, MA, USA). This system allows off-line analysis using digital calipers at a sweep speed of 200 mm/ms. Stimulation was performed on a commercially available cardiac stimulator (Micropace EPS cardiac stimulator, Micropace Pty Ltd., Canterbury, Australia).

For consistency, the placement of the atrial tissue was oriented in the same cranial-caudal and medial–lateral orientation with the epicardial surface in contact with the electrodes. A nylon weighting harp was placed over the tissue to improve contact with underlying electrodes. Stimulation of the atrial tissue was performed from two corners of the plaque (corner 1: inferior LA appendage (LAA), corner 2: LA free wall (LAFW)) at twice the capture threshold using a pulse width. A glass micropipette was inserted into the endocardial surface, and the intracellular membrane potential was recorded at alternate regions during atrial pacing from the stimulation sites at opposite corners of the MEA.

### 4.4. Atrial Refractoriness 

Refractoriness of the tissue was evaluated from the two corners of the plaque as outlined previously using an eight-beat (S1) stimuli drive train followed by a premature (S2) stimulus delivered in 10 ms decrements from an initial coupling interval of 100 ms. An effective refractory period (ERP) was defined as the longest S1–S2 interval failing to propagate an electrical impulse. This was repeated at 4 pacing cycle lengths (100, 200, 300, and 400 ms). ERP measurements were conducted thrice during each cycle length and averaged. We did not observe any episodes of spontaneous atrial arrhythmias (defined as >2 s) during standard S1-S2 ERP testing.

### 4.5. Atrial Conduction Analyses 

Conduction was assessed during constant capture at each cycle length using the local activation time maps. Activation maps were generated offline using semi-automated custom designed software to determine mean conduction velocity (CV) and conduction heterogeneity index (CHI). The impact of premature extra-stimuli was also evaluated to determine the effects of functional conduction abnormalities and performed by evaluating the shortest coupled extra-stimulus that resulted in a propagated response. Each annotation was manually verified by annotating the local activation time to the maximum deviation of the largest amplitude from the baseline on bipolar electrograms. CV was calculated from local vectors within each triangle of electrodes, while CHI was assessed using established phase-mapping techniques as previously described [60]. In brief, the phase distribution was obtained by calculating the largest difference in activation between every 4 adjacent electrodes. Absolute conduction phase delay was established through subtracting the 5th from 95th percentile of the phase distribution (P5-95), which was then divided by the median (P50) to derive the CHI. Total activation time (TAT) was defined as the longest conduction time in a given annotated map.

### 4.6. Atrial Histology

The left atrial tissue was fixed in 10% formalin before being wax embedded following standard routine procedures. Transverse sections (6 μm) were cut (Lecia RM2235 Rotary Microtome), mounted on to albumin-coated slides, and stained with Masson’s trichrome to determine the extent of fibrosis (blue staining) and the presence of inflammatory infiltrates with H&E staining. Histological slides were scanned at 40x magnification using the NanoZoomer Digital Pathology System (Hamamatsu Photonics, Hamamatsu, Japan). Five fields at 800× magnification were randomly selected per atrium, and images were exported, and myocyte cross-sectional diameter (100 cells in total per atrium) were independently analyzed by a blinded observer using the ruler function on NDP view 2 (Hamamatsu Photonics, Hamamatsu, Japan). Images were processed with Adobe Photoshop CC ver. 14.1.2 (Adobe Systems, San Jose, CA, USA) in accordance to published protocols [61], before the pixel content of staining for each atrium was measured relative to the total tissue area using ImageJ (ver. 1.7.0; NIH, Bethesda, MD, USA) with a batch macro following background subtraction.

Left atrial sections were deparaffinized using xylene and rehydrated. Slides were quenched for endogenous peroxidase with hydrogen peroxide–methanol solution for 30 min and antigen retrieved by citrate buffer (pH 6). Non-specific proteins were blocked with 3% normal horse serum for 20 min. Antibodies against Cx43 (Abcam, ab235585), Iba-1 (Abcam, ab5076), MMP-2 (Abcam, ab235167, Cambridge, UK), TIMP-1 (Abcam, ab216432), and TGFβ1 (Abcam, ab215715) were added to sections and incubated overnight at room temperature. Biotinylated anti-rabbit or anti-goat secondary (Vector Lab, BA-1000 and BA-9500) was applied to the respective sections for 60 min at room temperature, followed by streptavidin-conjugated peroxidase tertiary (ThermoFisher, 21127, Waltham, MA, USA) for 60 min at room temperature. Then, sections were visualized using diaminobenzidine tetrahydrochloride (DAB), washed, counterstained with hematoxylin, dehydrated, cleared, and mounted on glass coverslips. Immunostained sections were scanned at 20× magnification for blinded quantification using ImagePro Premium v9.1 (Media Cybernetics, Rockville, MD, USA) and expressed as proportion relative to the total myocardium area.

### 4.7. Multiplex Enzyme-Linked Immunoassay 

Blood samples were collected immediately before heart excision in 4 mL EDTA tubes with 1 mL heparinized syringes with a 21G needle via cardiac puncture in the left ventricle. Tubes were centrifuged for 10 min at 1500× *g* at 4 °C, and the plasma was transferred into 1.5 mL microcentrifuge tubes and stored at −20 °C. Plasma samples were analyzed for peptide levels of matrix metalloproteinase (MMP)-2 and -3, pro-MMP-9, tissue inhibitor of metalloproteinase-1 (TIMP-1), transforming growth factor beta-1 (TGFβ1), interleukin-6 (IL-6), C-reactive protein (CRP), tumor necrosis factor alpha (TNFα), intercellular adhesion molecule-1 (ICAM-1), and vascular cell adhesion molecule-1 (VCAM-1) using multiplex sandwich ELISA arrays (Custom Quantibody Array, Raybiotech, GA, USA) according to the manufacturer’s instructions. Circulating CRP and IL-6 measurements across groups were consistently higher and lower than the limits of detection respectively and were excluded from the analyses.

### 4.8. Statistical Analysis 

All data are presented as means ± standard error of mean (SEM). Two-way ANOVA was conducted to determine differences in age and HCM mutation contributions on animal characteristics, histology, immunohistochemistry, and biomarkers using GraphPad Prism 6 (GraphPad Software Inc., San Diego, CA, USA). Where significant, Tukey multiple comparisons were conducted. General linear model analysis of variance (GLM ANOVA) was used to determine significant differences of age and HCM mutation contributions on electrophysiological parameters as appropriate using PASW Statistics 18 (IBM Corp., Chicago, IL, USA). Statistical significance was established at *p* < 0.05.

## Figures and Tables

**Table 1:** Animal characteristics and heart chamber assessment.

	30 Weeks Old		50 Weeks Old	
	WT	HCM	WT	HCM
Body weight (g)	31.3 ± 1.2	30.3 ± 1.0	30.3 ± 0.3	31.3 ± 0.5
Heart rate (bpm)	295.5 ± 14.9	254.9 ± 20.4	268.7 ± 7.3	273.8 ± 12.4
Systolic blood pressure (mmHg)	94.5 ± 2.8	95.3 ± 1.5	86.4 ± 1.6 ǂ	85.2 ± 1.7 ǂ
LA mass (mg)	3.9 ± 0.4	6.8 ± 0.9 *	3.7 ± 0.1	8.0 ± 2.0 *
RA mass (mg)	4.1 ± 0.4	5.3 ± 0.6 *	3.6 ± 0.3	4.4 ± 0.5 *
LV wall thickness (mm)	1.2 ± 0.1	1.5 ± 0.1 *	1.2 ± 0.1	1.7 ± 0.1 *
Septal wall thickness (mm)	1.0 ± 0.1	1.3 ± 0.1 *	0.9 ± 0.1	1.4 ± 0.1 *

* *p* < 0.05 vs. age-matched wild-type (WT), ǂ *p* < 0.05 vs. young strain-matched mice.

## Data Availability

The authors declare that all other data supporting the findings of this study are available within the paper. Any additional information is available upon reasonable request to the corresponding author.

## Abbreviations

AF	Atrial fibrillation
APD	Action potential duration
CV	Conduction velocity
CHI	Conduction heterogeneity index
CRP	C-reactive protein
Cx43	Connexin 43
DAB	Diaminobenzidine tetrahydrochloride
ERP	Effective refractory period
GLM ANOVA	General linear model analysis of variance
HCM	Hypertrophic cardiomyopathy
H&E	Hematoxylin and eosin
Iba-1	Ionized calcium binding adaptor molecule 1
ICAM-1	Intercellular Adhesion Molecule 1
IL-6	Interleukin-6
LA	Left atria
LAA	Left atrial appendage
LAFW	Left atrial free wall
LV	Left ventricle
MEA	Multi-electrode array
MMP	Matrix metalloproteinase
MT	Masson’s trichrome
MYBPC3	Myosin-binding protein C3
MYH7	Myosin heavy chain 7
RA	Right atria
SEM	Standard error of the mean
TAT	Total activation time
TIMP	Tissue inhibitor of metalloproteinase
TGFβ1	Transforming growth factor beta 1
TNFα	Tumor necrosis factor alpha
TnI	Troponin I
VCAM-1	Vascular cell adhesion molecule 1
WT	Wild-type

## References

[1] Maron B.J. (2018). Clinical course and management of hypertrophic cardiomyopathy. N. Engl. J. Med..

[2] Semsarian C., Ingles J., Maron M.S., Maron B.J. (2015). New perspectives on the prevalence of hypertrophic cardiomyopathy. J. Am. Coll. Cardiol..

[3] Canepa M., Fumagalli C., Tini G., Vincent-Tompkins J., Day S.M., Ashley E.A., Mazzarotto F., Ware J.S., Michels M., Jacoby D. (2020). Temporal trend of age at diagnosis in hypertrophic cardiomyopathy: An analysis of the International Sarcomeric Human Cardiomyopathy Registry. Circ. Heart Fail..

[4] Bagnall R.D., Weintraub R.G., Ingles J., Duflou J., Yeates L., Lam L., Davis A.M., Thompson T., Connell V., Wallace J. (2016). A prospective study of sudden cardiac death among children and young adults. N. Engl. J. Med..

[5] Ho C.Y., Day S.M., Ashley E.A., Michels M., Pereira A.C., Jacoby D., Cirino A.L., Fox J.C., Lakdawala N.K., Ware J.S. (2018). Genotype and lifetime burden of disease in hypertrophic cardiomyopathy: Insights from the sarcomeric human cardiomyopathy registry (SHaRe). Circulation.

[6] Lee S.-E., Park J.-K., Uhm J.-S., Kim J.Y., Pak H.-N., Lee M.-H., Joung B. (2017). Impact of atrial fibrillation on the clinical course of apical hypertrophic cardiomyopathy. Heart.

[7] Alphonse P., Virk S., Collins J., Campbell T., Thomas S.P., Semsarian C., Kumar S. (2021). Prognostic impact of atrial fibrillation in hypertrophic cardiomyopathy: A systematic review. Clin. Res. Cardiol..

[8] Guttmann O.P., Rahman M.S., O’Mahony C., Anastasakis A., Elliott P.M. (2014). Atrial fibrillation and thromboembolism in patients with hypertrophic cardiomyopathy: Systematic review. Heart.

[9] Hindricks G., Potpara T., Dagres N., Arbelo E., Bax J.J., Blomström-Lundqvist C., Boriani G., Castella M., Dan G.-A., Dilaveris P.E. (2021). 2020 ESC guidelines for the diagnosis and management of atrial fibrillation developed in collaboration with the European Association for Cardio-Thoracic Surgery (EACTS): The task force for the diagnosis and management of atrial fibrillation of the European Society of Cardiology (ESC) developed with the special contribution of the European Heart Rhythm Association (EHRA) of the ESC. Eur. Heart J..

[10] Zoni-Berisso M., Lercari F., Carazza T., Domenicucci S. (2014). Epidemiology of atrial fibrillation: European perspective. Clin. Epidemiol..

[11] Rowin E.J., Hausvater A., Link M.S., Abt P., Gionfriddo W., Wang W., Rastegar H., Estes N.A.M., Maron M.S., Maron B.J. (2017). Clinical profile and consequences of atrial fibrillation in hypertrophic cardiomyopathy. Circulation.

[12] Siontis K.C., Geske J.B., Ong K., Nishimura R.A., Ommen S.R., Gersh B.J. (2014). Atrial fibrillation in hypertrophic cardiomyopathy: Prevalence, clinical correlations, and mortality in a large high-risk population. J. Am. Heart Assoc..

[13] Ball J., Carrington M.J., McMurray J.J.V., Stewart S. (2013). Atrial fibrillation: Profile and burden of an evolving epidemic in the 21st century. Int. J. Cardiol..

[14] Goldberger J.J., Arora R., Green D., Greenland P., Lee D.C., Lloyd-Jones D.M., Markl M., Ng J., Shah S.J. (2015). Evaluating the atrial myopathy underlying atrial fibrillation: Identifying the arrhythmogenic and thrombogenic substrate. Circulation.

[15] Tsoutsman T., Chung J., Doolan A., Nguyen L., Williams I.A., Tu E., Lam L., Bailey C.G., Rasko J.E.J., Allen D.G. (2006). Molecular insights from a novel cardiac troponin I mouse model of familial hypertrophic cardiomyopathy. J. Mol. Cell. Cardiol..

[16] Lim W.-W., Baumert M., Neo M., Kuklik P., Ganesan A.N., Lau D.H., Tsoutsman T., Semsarian C., Sanders P., Saint D.A. (2016). Slowed atrial and atrioventricular conduction and depressed HRV in a murine model of hypertrophic cardiomyopathy. Clin. Exp. Pharmacol. Physiol..

[17] Bonda T.A., Szynaka B., Sokołowska M., Dziemidowicz M., Winnicka M.M., Chyczewski L., Kamiński K.A. (2016). Remodeling of the intercalated disc related to aging in the mouse heart. J. Cardiol..

[18] Wijffels M.C., Kirchhof C.J., Dorland R., Allessie M.A. (1995). Atrial fibrillation begets atrial fibrillation. A study in awake chronically instrumented goats. Circulation.

[19] Morillo C.A., Klein G.J., Jones D.L., Guiraudon C.M. (1995). Chronic rapid atrial pacing. Structural, functional, and electrophysiological characteristics of a new model of sustained atrial fibrillation. Circulation.

[20] Li D., Fareh S., Leung T.K., Nattel S. (1999). Promotion of atrial fibrillation by heart failure in dogs: Atrial remodeling of a different sort. Circulation.

[21] Sanders P., Morton J.B., Davidson N.C., Spence S.J., Vohra J.K., Sparks P.B., Kalman J.M. (2003). Electrical remodeling of the atria in congestive heart failure. Circulation.

[22] Shinagawa K., Shi Y.-F., Tardif J.-C., Leung T.-K., Nattel S. (2002). Dynamic nature of atrial fibrillation substrate during development and reversal of heart failure in dogs. Circulation.

[23] Alasady M., Shipp N.J., Brooks A.G., Lim H.S., Lau D.H., Barlow D., Kuklik P., Worthley M.I., Roberts-Thomson K.C., Saint D.A. (2013). Myocardial infarction and atrial fibrillation: Importance of atrial ischemia. Circ. Arrhythm. Electrophysiol..

[24] Mahajan R., Lau D.H., Brooks A.G., Shipp N.J., Manavis J., Wood J.P.M., Finnie J.W., Samuel C.S., Royce S.G., Twomey D.J. (2015). Electrophysiological, electroanatomical, and structural remodeling of the atria as consequences of sustained obesity. J. Am. Coll. Cardiol..

[25] Iwasaki Y.-K., Kato T., Xiong F., Shi Y.-F., Naud P., Maguy A., Mizuno K., Tardif J.-C., Comtois P., Nattel S. (2014). Atrial fibrillation promotion with long-term repetitive obstructive sleep apnea in a rat model. J. Am. Coll. Cardiol..

[26] Lau D.H., Mackenzie L., Kelly D.J., Psaltis P.J., Brooks A.G., Worthington M., Rajendram A., Kelly D.R., Zhang Y., Kuklik P. (2010). Hypertension and atrial fibrillation: Evidence of progressive atrial remodeling with electrostructural correlate in a conscious chronically instrumented ovine model. Heart Rhythm.

[27] Lau D.H., Psaltis P.J., Mackenzie L., Kelly D.J., Carbone A., Worthington M., Nelson A.J., Zhang Y., Kuklik P., Wong C.X. (2011). Atrial remodeling in an ovine model of anthracycline-induced nonischemic cardiomyopathy: Remodeling of the same sort. J. Cardiovas. Electrophysiol..

[28] Sutanto H., Cluitmans M.J.M., Dobrev D., Volders P.G.A., Bébarová M., Heijman J. (2020). Acute effects of alcohol on cardiac electrophysiology and arrhythmogenesis: Insights from multiscale in silico analyses. J. Mol. Cell. Cardiol..

[29] Chen Q., Yi Z., Cheng J. (2018). Atrial fibrillation in aging population. Aging Med..

[30] Cherry E.M., Fenton F.H. (2011). Effects of boundaries and geometry on the spatial distribution of action potential duration in cardiac tissue. J. Theor. Biol..

[31] Bishop M.J., Connolly A., Plank G. (2014). Structural heterogeneity modulates effective refractory period: A mechanism of focal arrhythmia initiation. PLoS ONE.

[32] Geisterfer-Lowrance A.A., Christe M., Conner D.A., Ingwall J.S., Schoen F.J., Seidman C.E., Seidman J.G. (1996). A mouse model of familial hypertrophic cardiomyopathy. Science.

[33] McConnell B.K., Fatkin D., Semsarian C., Jones K.A., Georgakopoulos D., Maguire C.T., Healey M.J., Mudd J.O., Moskowitz I.P., Conner D.A. (2001). Comparison of two murine models of familial hypertrophic cardiomyopathy. Circ. Res..

[34] Prabhakar R., Boivin G.P., Grupp I.L., Hoit B., Arteaga G., Solaro R.J., Wieczorek D.F. (2001). A familial hypertrophic cardiomyopathy alpha-tropomyosin mutation causes severe cardiac hypertrophy and death in mice. J. Mol. Cell. Cardiol..

[35] Vikstrom K.L., Factor S.M., Leinwand L.A. (1996). Mice expressing mutant myosin heavy chains are a model for familial hypertrophic cardiomyopathy. Mol. Med..

[36] Dharmaprani D., Schopp M., Kuklik P., Chapman D., Lahiri A., Dykes L., Xiong F., Aguilar M., Strauss B., Mitchell L. (2019). Renewal theory as a universal quantitative framework to characterize phase singularity regeneration in mammalian cardiac fibrillation. Circ. Arrhythm. Electrophysiol..

[37] Dharmaprani D., Jenkins E., Aguilar M., Quah J.X., Lahiri A., Tiver K., Mitchell L., Kuklik P., Meyer C., Willems S. (2021). M/M/Infinity birth-death processes–A quantitative representational framework to summarize and explain phase singularity and wavelet dynamics in atrial fibrillation. Front. Physiol..

[38] Van Hunnik A., Zeemering S., Podziemski P., Kuklik P., Kuiper M., Verheule S., Schotten U. (2021). Bi-atrial high-density mapping reveals inhibition of wavefront turning and reduction of complex propagation patterns as main antiarrhythmic mechanisms of vernakalant. Europace.

[39] Kistler P.M., Sanders P., Fynn S.P., Stevenson I.H., Spence S.J., Vohra J.K., Sparks P.B., Kalman J.M. (2004). Electrophysiologic and electroanatomic changes in the human atrium associated with age. J. Am. Coll. Cardiol..

[40] Osadchii O.E. (2017). Role of abnormal repolarization in the mechanism of cardiac arrhythmia. Acta Physiol..

[41] Spach M.S., Boineau J.P. (1997). Microfibrosis produces electrical load variations due to loss of side-to-side cell connections; A major mechanism of structural heart disease arrhythmias. Pacing Clin. Electrophysiol..

[42] Roldán V., Marín F., Gimeno J.R., Ruiz-Espejo F., González J., Feliu E., García-Honrubia A., Saura D., de la Morena G., Valdés M. (2008). Matrix metalloproteinases and tissue remodeling in hypertrophic cardiomyopathy. Am. Heart J..

[43] Bi X., Yang C., Song Y., Yuan J., Cui J., Hu F., Qiao S. (2021). Matrix metalloproteinases increase because of hypoperfusion in obstructive hypertrophic cardiomyopathy. Ann. Thorac. Surg..

[44] Meschiari C.A., Ero O.K., Pan H., Finkel T., Lindsey M.L. (2017). The impact of aging on cardiac extracellular matrix. Geroscience.

[45] Xu J., Cui G., Esmailian F., Plunkett M., Marelli D., Ardehali A., Odim J., Laks H., Sen L. (2004). Atrial extracellular matrix remodeling and the maintenance of atrial fibrillation. Circulation.

[46] Mukherjee R., Akar J.G., Wharton J.M., Adams D.K., McClure C.D., Stroud R.E., Rice A.D., DeSantis S.M., Spinale F.G., Gold M.R. (2013). Plasma profiles of matrix metalloproteinases and tissue inhibitors of the metalloproteinases predict recurrence of atrial fibrillation following cardioversion. J. Cardiovasc. Transl. Res..

[47] Tsoutsman T., Wang X., Garchow K., Riser B., Twigg S., Semsarian C. (2013). CCN2 Plays a key role in extracellular matrix gene expression in severe hypertrophic cardiomyopathy and heart failure. J. Mol. Cell. Cardiol..

[48] Teekakirikul P., Eminaga S., Toka O., Alcalai R., Wang L., Wakimoto H., Nayor M., Konno T., Gorham J.M., Wolf C.M. (2010). Cardiac fibrosis in mice with hypertrophic cardiomyopathy is mediated by non-myocyte proliferation and requires Tgf-β. J. Clin. Investig..

[49] Harada M., Van Wagoner D.R., Nattel S. (2015). Role of inflammation in atrial fibrillation pathophysiology and management. Circ. J..

[50] Heijman J., Muna A.P., Veleva T., Molina C.E., Sutanto H., Tekook M., Wang Q., Abu-Taha I.H., Gorka M., Künzel S. (2020). Atrial myocyte NLRP3/CaMKII nexus forms a substrate for postoperative atrial fibrillation. Circ. Res..

[51] Zhang J., Alcaide P., Liu L., Sun J., He A., Luscinskas F.W., Shi G.-P. (2011). Regulation of endothelial cell adhesion molecule expression by mast cells, macrophages, and neutrophils. PLoS ONE.

[52] Schultz C.D., Rangneker G., Lim H.S., Fraudeau A., Young G., Roberts-Thomson K., John B., Worthley M., Sanders P., Willoughby S.R. (2014). Characterization of thrombogenic, endothelial and inflammatory markers in supraventricular tachycardia: A study in patients with structurally normal hearts. Clin. Exp. Pharmacol. Physiol..

[53] Kitz S., Fonfara S., Hahn S., Hetzel U., Kipar A. (2019). Feline hypertrophic cardiomyopathy: The consequence of cardiomyocyte-initiated and macrophage-driven remodeling processes?. Vet. Pathol..

[54] Willeit K., Pechlaner R., Willeit P., Skroblin P., Paulweber B., Schernthaner C., Toell T., Egger G., Weger S., Oberhollenzer M. (2017). Association between vascular cell adhesion molecule 1 and atrial fibrillation. JAMA Cardiol..

[55] Vaidya K., Semsarian C., Chan K.H. (2017). Atrial fibrillation in hypertrophic cardiomyopathy. Heart Lung Circ..

[56] Pujadas S., Vidal-Perez R., Hidalgo A., Leta R., Carreras F., Barros A., Bayes-Genis A., Subirana M.T., Pons-Llado G. (2010). Correlation between myocardial fibrosis and the occurrence of atrial fibrillation in hypertrophic cardiomyopathy: A cardiac magnetic resonance imaging study. Eur. J. Radiol..

[57] Gruver E.J., James Gruver E., Fatkin D., Alfred Dodds G., Kisslo J., Maron B.J., Seidman J.G., Seidman C.E. (1999). Familial hypertrophic cardiomyopathy and atrial fibrillation caused by Arg663His beta–cardiac myosin heavy chain mutation. Am. J. Cardiol..

[58] Thanigaimani S., Brooks A.G., Kuklik P., Twomey D.J., Franklin S., Noschka E., Chapman D., Pathak R.K., Mahajan R., Sanders P. (2017). Spatiotemporal characteristics of atrial fibrillation electrograms: A novel marker for arrhythmia stability and termination. J. Arrhythm..

[59] Dinshaw L., Münkler P., Schäffer B., Klatt N., Jungen C., Dickow J., Tamenang A., Schleberger R., Pecha S., Pinnschmidt H. (2021). Ablation of atrial fibrillation in patients with hypertrophic cardiomyopathy: Treatment strategy, characteristics of consecutive atrial tachycardia and long-term outcome. J. Am. Heart Assoc..

[60] Neo M., Morris D.G., Kuklik P., Lau D.H., Dimitri H., Lim W.-W., Sanders P., Saint D.A. (2016). Simultaneous conduction mapping and intracellular membrane potential recording in isolated atria. Can. J. Physiol. Pharmacol..

[61] Dahab G.M., Kheriza M.M., El-Beltagi H.M., Fouda A.-M.M., El-Din O.A.S. (2004). Digital quantification of fibrosis in liver biopsy sections: Description of a new method by photoshop software. J. Gastroenterol. Hepatol..

